# *Boswellia dalzielii*-Mediated Silver Nanoparticles Inhibited Acute Myeloid Leukemia (AML) Kasumi-1 Cells by Inducing Cell Cycle Arrest

**DOI:** 10.1155/2020/8898360

**Published:** 2020-09-22

**Authors:** Ismail Abiola Adebayo, Adamu Ibrahim Usman, Fatimah Bukola Shittu, Noor Zafirah Ismail, Hasni Arsad, Taoheed Kolawole Muftaudeen, Mohammed Razip Samian

**Affiliations:** ^1^Integrative Medicine Cluster, Advanced Medical and Dental Institute, Universiti Sains Malaysia, 13200 Bertam, Kepala Batas, Penang, Malaysia; ^2^Department of Microbiology and Immunology, Faculty of Biomedical Sciences, Kampala International University, Western Campus, P.O. Box 71, Ishaka, Bushenyi, Uganda; ^3^Department of Physics, Faculty of Science, Federal University Kashere, Gombe, Gombe State, Nigeria; ^4^Science Laboratory Technology Department, School of Applied Sciences, The Federal Polytechnic, Offa, Kwara, Nigeria; ^5^Department of Biological Sciences, Faculty of Computing and Applied Sciences, Baze University, Abuja, Nigeria; ^6^School of Biological Sciences, Universiti Sains Malaysia, 11800 George Town, Penang, Malaysia

## Abstract

**Background:**

Acute myeloid leukemia (AML) persists to be a major health problem especially among children as effective chemotherapy to combat the disease is yet to be available. *Boswellia dalzielii* is a well-known herb that is traditionally used for treatment and management of many diseases including degenerative diseases. In this study, silver nanoparticles were synthesized from the phytochemicals of *B. dalzielii* stem bark aqueous extract. The silver nanoparticles were characterized by carrying out Fourier Transform Infrared (FTIR) spectroscopy, Energy Filtered Scanning Electron Microscopy (FESEM), X-ray diffraction, and Dynamic Light Scattering (DLS) analyses. Antioxidant capacity of the nanoparticles was evaluated using 2,2-diphenyl-1-picrylhydrazyl (DPPH) assay, and the antiproliferative effect of the nanoparticles on Kasumi-1 leukemia cells was investigated using PrestoBlue assay. Flow cytometry analysis was performed to observe the effect of the nanoparticles on the leukemia cell cycle progression.

**Results:**

Our findings revealed that the synthesized silver nanoparticles were formed from electrons of the plant phytochemicals which include aromatic compounds, ethers, and alkynes. FESEM analysis revealed that the sizes of the nanoparticles range from 12 nm to 101 nm; however, DLS analysis estimated a larger average size of the nanoparticles (108.3 nm) because it measured the hydrodynamic radii of the nanoparticles. The zeta potential of the nanoparticles was −16 nm, and the XRD pattern of the nanoparticles has distinct peaks at 38.02°, 42.94°, 64.45°, 77.20°, and 81.47°, which is typical of face-centered cubic (fcc) structure of silver. The Trolox Equivalence Antioxidant Capacity (TEAC) of the nanoparticles was estimated to be 300.91 *μ*M Trolox/mg silver nanoparticles. The nanoparticles inhibited Kasumi-1 cell proliferation. The half minimal inhibitory concentrations (IC50s) that inhibited Kasumi-1 cell proliferation are 49.5 *μ*g/ml and 13.25 *μ*g/ml at 48 and 72 hours, respectively. The nanoparticles induced cell cycle arrest in the Kasumi-1 cells at S (5% increase) and G2/M (3% increase) phases.

**Conclusion:**

The nanoparticles synthesized from the stem bark extract of *B. dalzielii* inhibit the growth of Kasumi-1 leukemia cells by activating cell cycle arrest; thus, they are potential antileukemic agents.

## 1. Introduction

Acute myeloid leukemia (AML) continues to be a serious health problem as effective chemotherapy to combat the disease is still lacking [1]. The disease is responsible for about 15–20% of acute leukemia among children, and it accounts for more than half of the pediatric leukemia patients that died [2]. Even though the current available chemotherapy has greatly improved the efficiency of the treatment and management of AML which resulted in long-term survival in about 65% of AML pediatric patients, the chemotherapy has many serious side effects which include the following: (a) some patients do not respond to the chemotherapy treatment; (b) half of the patients that respond to the chemotherapy treatment do experience relapse afterwards and eventually die [2–4]. Therefore, there is a burning need for the discovery of more efficient new chemotherapy for the treatment of AML.

*Boswellia dalzielii* is a plant that is popularly used for medicinal purposes in some West African countries such as Ghana, Benin, Togo, Burkina Faso, Northern Nigeria, Cameroon, and Northern Ivory Coast [5, 6]. The plant is traditionally used to treat several diseases and illnesses which include digestive disorders, skin diseases, tuberculosis, nervous disorders, diarrheal disease, inflammation, analgesia, pyretic fever, and gingivitis [5, 6]. Experimental findings also established that the plant has antinociceptive properties [5]. The plant has many electron-rich compounds in abundance that can donate their electrons to reduce silver to synthesize silver nanoparticles [5]. This makes the plant a good candidate for nanoparticle production [5]. The electron-rich compounds the plant has include phenolics, flavonoids, alkaloids, glycosides, triterpenoids, carbohydrates, saponins, gallic acid, anthraquinones, protocatechuic acids, and *β*-sitosterol to mention but a few [5, 7–9].

In recent times, nanoparticle synthesis and applications have gained tremendous interest and attention of researchers all over the world because of their potential usefulness in many fields such as medicine, biology, pharmaceutical industry, and textile industry [10, 11]. Physical, chemical, and biological (green) methods are the three major methods of synthesizing nanoparticles [12]. However, the disadvantages of the physical and chemical methods, such as pollution of environment, high cost, and longer period of reaction, make the green biological method stands out as the most preferred one [13, 14]. In addition, the biological method is green synthesis, which is eco-friendly, easy, nontoxic, cheap, and faster way of synthesizing the nanoparticles [12]. In green synthesis, plants, microorganisms, and animal materials are the sources of chemical compounds (metabolites), which serve as reducing agents to reduce silver to nanoparticles and stabilize the nanoparticles [15].

Silver nanoparticles synthesized from phytochemicals (plant compounds) have been found to have antimicrobial, anticancer, antioxidant, and antidiabetic properties [15]. For instance, silver nanoparticles synthesized from the phytochemicals of *Alternanthera sessilis, Dendrophthoe falcata, Albizia adianthifolia, Morinda citrifolia, Detarium microcarpum,* and *Artemisia turcomanica* were reported to have antiproliferative effects on the growth of prostate cancer (PC3), breast cancer (MCF7), lung cancer (A549), cervical cancer (HeLa), pancreatic cancer, and gastric cancer (AGS) cells, respectively [14, 16–20]. Hence, owing to the fact that there is a pressing need for effective therapy for leukemia, in this study, we investigated the potency of the silver nanoparticles synthesized from *B. dalzielii* stem bark metabolites to inhibit the growth and proliferation of AML cell line, Kasumi-1 cells.

## 2. Materials and Methods

### 2.1. Plant Extraction and Silver Nanoparticle Synthesis

The stem bark of the plant *B. dalzielii* was collected from the Azare forest in the Katagum Local Government Area of Bauchi State of Nigeria. The identity of the plant sample was authenticated by qualified expert in the Biological Sciences Department of Ahmadu Bello University, Nigeria. Phytochemicals were extracted from the plant sample by soaking 50 g of the sample powdered form in 250 ml of water, and the mixture was incubated for five days with continuous shaking. The extraction was done one more time using the same procedure with the same powder. The mixture was filtered, and the liquid fraction (water extract) containing the electron-rich metabolites was dried. Five grams of the plant extract was resuspended in water (250 ml), and the liquid extract was added to 750 ml of 5 mM silver nitrate (Sigma-Aldrich, Darmstadt, Germany) at room temperature. The reaction in the mixture lasted for 1.5 hours during which the silver nanoparticle was being synthesized. The reaction was observed every thirty minutes using the Ultraviolet- (UV) visible (Vis) spectrometer (AK36735, Perkin Elmer, Waltham, Massachusetts, USA) to measure the mixture absorbance within the wavelength range of 400–700 nm.

### 2.2. Characterization of the Silver Nanoparticles

The functional groups of the compounds in the used plant extract that reduced the silver to form the silver nanoparticles were identified using Fourier Transform Infrared (FTIR) spectroscopy (Perkin Elmer System 2000, Waltham, Massachusetts, USA). The morphology of the synthesized silver nanoparticles was observed using Energy Filtered Scanning Electron Microscopy (EFSEM) (Shimadzu UV-3600, Kyoto, Japan). The polydispersity index, hydrodynamic sizes, and zeta potential of the silver nanoparticles were determined using Dynamic Light Scattering (DLS) instruments (Zetasizer Nano ZS, Malvern Instruments, Ltd., Malvern, UK). The X-ray diffraction (XRD) analysis was performed on the nanoparticle using an X-ray diffractometer (Bruker AXS K. K. 3–9, Yokohama-shi, Kanagawa, Japan).

### 2.3. 1,1-Diphenyl-2-picrylhydrazyl (DPPH) Radical Scavenging Assay

The DPPH assay was carried out using a modification of the procedure described by [21]. Briefly, 150 *μ*l of 0.2 mM DPPH solution (Sigma-Aldrich, Darmstadt, Germany) was added to either 50 *μ*l of the synthesized silver nanoparticles or 50 *μ*l of varying concentrations (0, 30, 60, 120, 240, and 480 *μ*g/ml) of Trolox antioxidant solution (Acros Organics, Newton Dr, Carlsbad, CA, USA) in wells of a 96-well plate. The mixtures were mixed and incubated for 20 minutes in the dark. The absorbance values of the solutions were read at 517 nm. Percentage radical scavenging activity (RSA) for each sample solution (both Trolox and silver nanoparticles) was calculated using the following formula:

(1) $$\begin{array}{c} \text{RSA}\left(\%\right)=\left(\frac{\left[\text{BA}-\text{SA}\right]}{\text{BA}}\right)\times 100, \end{array}$$

where BA is the blank absorbance and SA is the sample absorbance (Trolox or synthesized AgNps).

The Trolox standard curve was plotted, and the Trolox Equivalence Antioxidant Capacity (TEAC) of the nanoparticles was then estimated from the standard curve.

### 2.4. Cell Culture Growth and Maintenance

The Kasumi-1 cells (Leibniz-Institute DSMZ, Braunschweig, Germany) were cultured and maintained in RPMI 1640 complete growth medium supplemented with 10% (v/v) fetal bovine serum (FBS) and 1% pen-strep (penicillin-streptomycin) antibiotics in a flask. The cells were subcultured after they have grown up to 80% confluence on the growth surface of the flask. The medium, FBS, antibiotics were obtained from Thermo Fisher Scientific (Waltham, Massachusetts, USA).

### 2.5. PrestoBlue Antiproliferative Assay

The inhibitory effect of the synthesized silver nanoparticles on AML Kasumi-1 cells was investigated using PrestoBlue antiproliferative assay according to the experimental procedure described by [21]. The cells (5000 cells/well) were seeded into the wells of a 96-well plate and treated with different concentrations of the silver nanoparticles (0–70 *μ*g/ml) for 48 and 72 hours. After the treatment, 10 *μ*l of PrestoBlue resazurin dye solution (Thermo Fisher Scientific, Waltham, MA, USA) was added to each well, and the plate was incubated in the dark for 1-2 hours. Then, absorbance values of each well were read at 600 nm (emission state) and 570 nm (excitation state) using spectrophotometer. The percentage cell viability for each concentration of the silver nanoparticles was calculated using the following formula:

(2) $$\begin{array}{c} \text{percentage cell viability}=\frac{\left(\text{SF}-\text{BF}\right)}{\left(\text{CF}-\text{BF}\right)}\times 100, \end{array}$$

where SF is the sample (cells treated with silver nanoparticles) fluorescence, BF is the blank fluorescence, and CF is the control (cells treated with DMSO only) fluorescence.

### 2.6. Flow Cytometry Cell Cycle Analysis

The cell cycle analysis was carried out using PI/RNase cell cycle analysis reagent (Invitrogen, Thermo Fisher Scientific, Carlsbad, CA, USA) based on the method suggested by the manufacturer of the reagent. The Kasumi-1 cells (2.5 × 10^5^ cell/well) in 3 ml were seeded in wells of 6-well plate. The cells were treated with silver nanoparticles (20 *μ*g/ml) for 72 hours. Then, the treated cells were harvested, and they were fixed with 70% ethanol by incubation in the fixing solution for 1-2 hours at 4°C. The fixed cells were washed with PBS, and they were resuspended in 0.5 ml of the PI/RNase staining solution. The cells were incubated in the staining solution for 15 minutes in the dark at room temperature. Then, they were analyzed by flow cytometer without delay.

### 2.7. Statistical Analysis

SPSS software (version 24) was employed for statistical analysis. The numerical values are presented as the mean ± SEM of 3-4 independent results. One-way ANOVA was used to determine the significance of the results, and only the results that are different at significant level of *p* < 0.05 are presented.

## 3. Results and Discussion

### 3.1. UV-Vis

**Figure 1:**
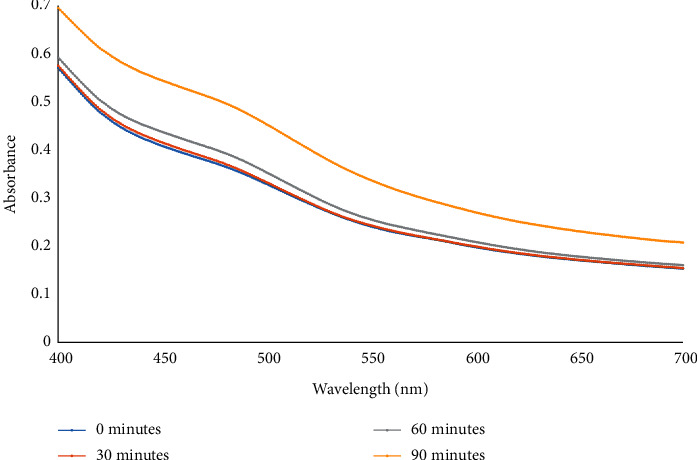
The UV-Vis spectra of the reaction mixture containing *B. dalzielii* silver nanoparticles (AgNps). The synthesis of the silver nanoparticles in the mixture was monitored using UV-Vis at 4 different time intervals (0, 30, 60, and 90 minutes). The spike peak observed at 480 nm signifies the surface plasmon resonance (SPR) of the synthesized silver nanoparticles. The absorbance of the reaction mixture with the nanoparticles increased as the reaction time increased.

The synthesis of the silver nanoparticles in the reaction mixture that contained the silver nitrate and the plant extract was periodically observed by UV-Vis spectroscopy, and the results are displayed in Figure 1. It was observed that a broad peak surfaced around the wavelength of 480 nm, and its intensity increased with time. The peak that was observed around 480 nm could be attributed to the surface plasmon resonance (SPR) of the nanoparticles that were being synthesized in the reaction mixture. The observation is in tandem with the SPRs that were similarly observed for other silver nanoparticles synthesized from natural sources [22, 23]. For instance, SPRs of the silver nanoparticles synthesized from cayenne pepper (*Capsicum frutescens*) and *Buddleja globosa* metabolites were observed at 480 nm in the reaction mixtures [23, 24]. The broadening of the peak (SPR) signifies that the synthesized nanoparticles are poly dispersed with different shapes and sizes [25].

### 3.2. FTIR Analysis of Silver Nanoparticles

**Figure 2:**
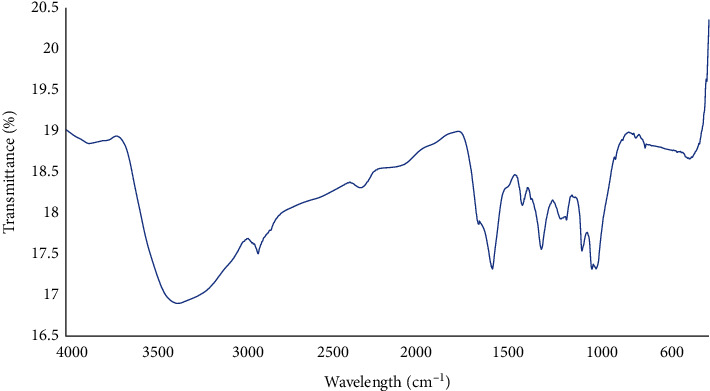
FTIR of *B. dalzielii* AgNps. The peaks identified in the nanoparticle solution represent the functional groups of the chemical compounds of *B. dalzielii* that reduced silver to form the nanoparticles.

The FTIR analysis of the synthesized silver nanoparticles was performed to unveil the functional groups of the plant extract which served as reductants for the synthesis of the silver nanoparticles. The FTIR spectrum of the nanoparticles has several peaks which could be attributed to many functional groups of some chemical compounds (Figure 2). The peak at 3371 nm corresponds to the O-H stretch of hydroxyl group of organic compounds [26]. The peaks that were found at 2926 nm, 1445 nm, 1231 nm, 1198 nm, 1054 nm, 1030 nm, 810 nm, and 757 nm could be attributed to the vibration of C-H stretch that is part of different functional groups of organic compounds such as the aromatic and ether compounds [26]. The peak at 2351 could be attributed to the –C≡C-triple bond stretch of the alkyne compounds [26]. The peak at 1677 nm indicates the possible presence of the stretch of –C=C- of alkene functional group and the stretch of –C=O- of carboxylic acid functional group. The peak at 1612 may be attributed to the stretches of –C-O- and –C=C- C of aromatic compounds. The peak found at 1336 nm may also be ascribed to the O-H stretch in phenol and tertiary alcohols [26].

### 3.3. Characterization of the Silver Nanoparticles

**Figure 3:**
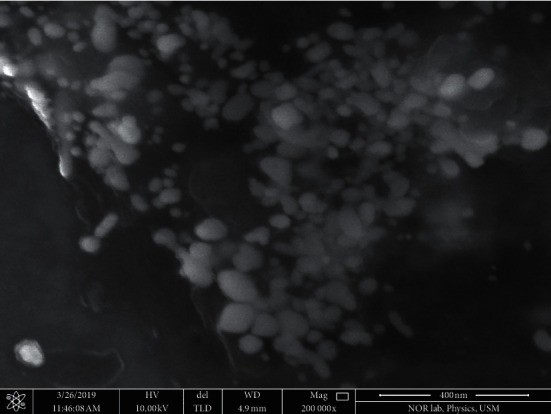
Scanning electron microscopic (SEM) view of *B. dalzielii* AgNps. The formed silver nanoparticles were viewed with scanning electron microscope to determine their shapes and sizes. Based on the microscopic picture, the nanoparticles have different shapes and sizes. Their shapes include rod, circular, spherical, and rectangular shapes. Their sizes ranged from 12 to 101 nm.

FESEM analysis of the silver nanoparticles revealed the morphologies and sizes of the nanoparticles (Figure 3). The nanoparticles appear aggregated, and they have different shapes which include circular, spherical, rod, and rectangular shapes (Figure 3). Also, the nanoparticles' sizes vary from 12 nm to 101 nm (Figure 3). Hence, the SEM results showed that the nanoparticles are heterogeneous with different sizes and morphologies, and this is not uncommon to silver nanoparticles that were synthesized from plants [27].

**Figure 4:**
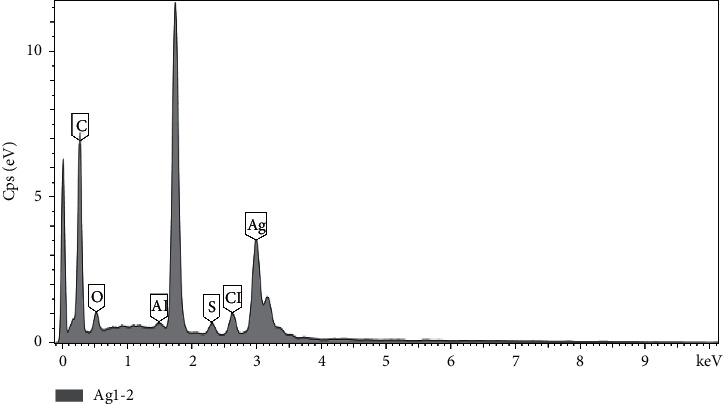
Energy-dispersive X-ray (EDX) spectra of *B. dalzielii* AgNps. The absorption peak found around 3 keV indicates the formation and presence of silver nanoparticles in the reaction mixture. As indicated in the result, other peaks found in the mixture represent the presence of chlorine, aluminum, sulfur, carbon, and oxygen.

The energy-dispersive X-ray (EDX) spectrum further affirms that the synthesis of silver nanoparticles occurring as an optical absorption peak was found at around 3 keV, which signals the presence of silver nanocrystals in the solution, because the peak represents surface plasmon resonance of the nanocrystals [28] (Figure 4). Other elements that were found at significant amounts in the silver nanoparticles solution include carbon, oxygen, aluminum, sulfur, and chlorine (Figure 4).

**Figure 5:**
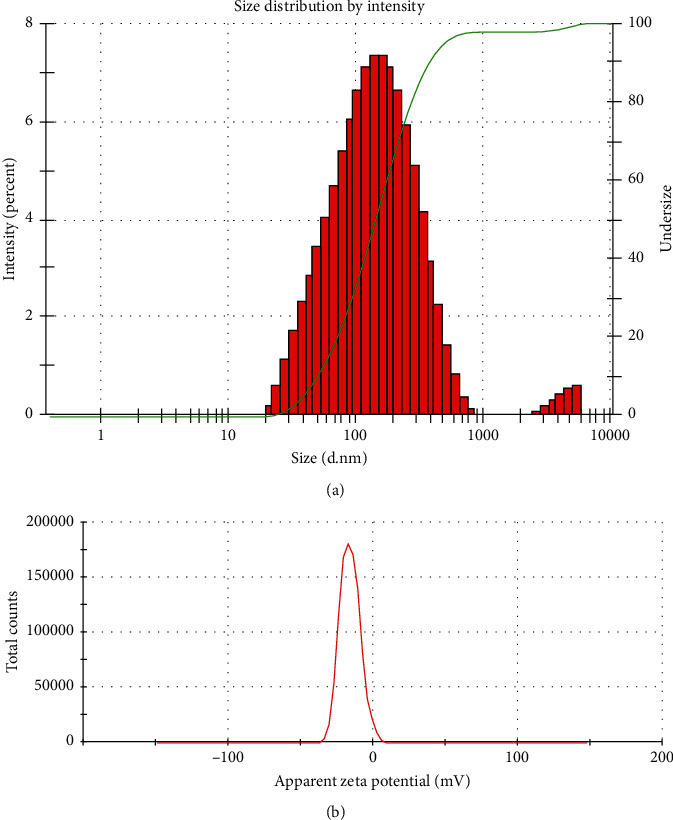
(a) Size distribution of *B. dalzielii* AgNps as analyzed by DLS. The poly disparity and sizes of the synthesized silver nanoparticles were observed using DLS. The result showed that the nanoparticles are poly dispersed and the average size of the nanoparticles is 108.3 nm. (b) Zeta potential graph of *B. dalzielii* AgNps. The stability of the synthesized silver nanoparticles was evaluated by their zeta potential. The zeta potential of the nanoparticles was found to be −16 mV which means the nanoparticles are relatively stable.

The particle size distribution of the synthesized nanoparticles was investigated using DLS, and it was revealed that the particles are highly poly dispersed with varying sizes (Figure 5(a)). The average diameter size of the nanoparticles is 108.3 nm. The poly disparity index is estimated to be 0.403 (Figure 5(a)). The sizes of the nanoparticles as estimated by DLS were expectedly larger compared to the result of SEM analysis because of the agglomeration of the synthesized silver nanoparticles [29]. Also, unlike SEM, the DLS measured the hydrodynamic radii of the silver nanoparticles which include the layers of the capping reducing agents from the plant extracts [29].

The silver nanoparticles have zeta potential value of −16 mV (Figure 5(b)) which falls within the range that is considered to be relatively stable (±10–20 mV) [30]. The negative value of the zeta potential indicates that there is repulsion among the particles which could also enhance the stability of the particles [31]. Supportively, the zeta potential value of the nanoparticles is also lower than some of the values reported for phytochemical-mediated synthesized silver nanoparticles which were considered to be stable and potent cytotoxins [29] (Figure 5(b)).

**Figure 6:**
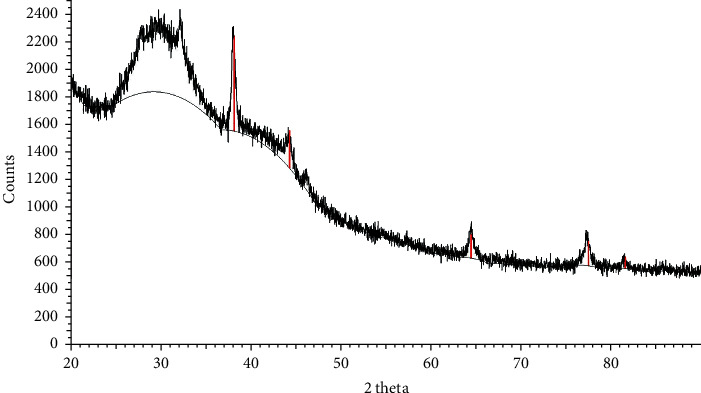
XRD pattern of *B. dalzielii* AgNps. The XRD pattern of the nanoparticles showed distinct peaks at 38.02°, 42.94°, 64.45°, 77.20°, and 81.47° in the range of 2*θ* which can be indexed to the (111), (200), (220), (311), and (222) crystallographic planes of face-centered cubic (fcc) structure of silver, respectively. Therefore, the XRD pattern agrees with the specifications stated by the Joint Committee on Powder Diffraction Standard (JPDS) file no. 04-0783 for metallic silver. Hence, the XRD pattern showed that the silver nanoparticles have crystalline structure.

X-ray diffraction (XRD) analysis was performed to determine and characterize the crystalline structure of the synthesized sliver nanoparticles.  Figure 6 shows the XRD pattern of the silver nanoparticles that were synthesized from *B. dalzielii*. The results revealed that the XRD pattern has distinct peaks at 38.02°, 42.94°, 64.45°, 77.20°, and 81.47° in the range of 2*θ* which can be indexed to the (111), (200), (220), (311), and (222) crystallographic planes of face-centered cubic (fcc) structure of silver, respectively [32]. The XRD pattern is also similar to the specifications reported by the Joint Committee on Powder Diffraction Standard (JPDS) file no. 04-0783 for a typical metallic silver [33]. Putting these together, the XRD pattern revealed that the synthesized silver nanoparticles are crystalline in nature.

### 3.4. Antioxidant Activity of the Synthesized Nanoparticles

**Figure 7:**
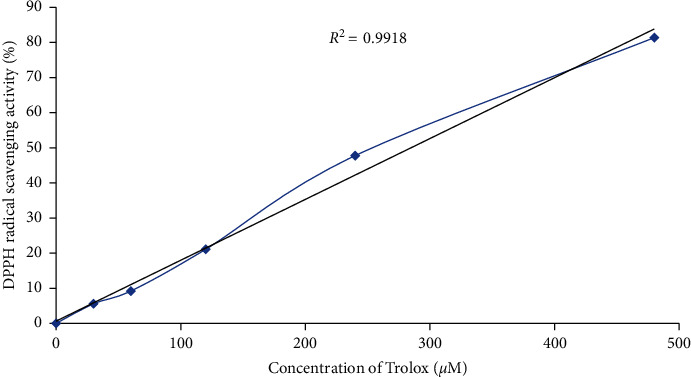
The Trolox standard curve used as standard for evaluating the antioxidant activity of *B. dalzielii* AgNps. The standard curve was created using DPPH assay, and the results showed that the antioxidant effect of the Trolox standard is directly proportional to its concentration as the quantified radical scavenging effect kept increasing when the concentration of Trolox was raised. Each value plotted is a mean of three independent replicates. The values are significantly different from one another at *p* < 0.05 as determined by one-way ANOVA.

In order to evaluate the biological activity and potential health benefit of the synthesized nanoparticles, their antioxidant capacity was evaluated using DPPH assay because antioxidants are generally helpful in management, prevention, and treatment of many diseases such as cancer, Alzheimer, inflammatory illness, and neurodegenerative diseases [34]. The antioxidant capacity of the nanoparticles was estimated as Trolox Equivalence Antioxidant Capacity (TEAC). The Trolox standard curve has the equation *y* = 0.1732 *x* + 0.645 with *R*^2^ value of 0.9918 (Figure 7). One milligram of the synthesized nanoparticles quenched 53.73% of the oxidant DPPH solution, and its TEAC value was calculated to be 300.91 ± 13.42 *μ*M Trolox/mg silver nanoparticles. The amount is considered significant when compared with the antioxidant capacities of other nanoparticles and medicinal plants [21, 35–37]. El-Rafie and Hamed [38] reported that *Terminalia catappa* extract-mediated silver nanoparticles had significant free radical scavenging effect and high antioxidant activity. Similarly, biogenic silver nanoparticles synthesized from *Pongamia pinnata* phytochemicals were proven to have free radical quenching activity and antioxidant potential [39]. Also, it was shown that silver nanoparticles biosynthesized from the chemical compounds of *Prosopis farcta* fruit possessed antioxidant activity [40]. Therefore, considering all these inferences, it could be said that the synthesized nanoparticles from the metabolites of the *B. dalzielii* extract have antioxidant effect.

### 3.5. Antiproliferative Effect of the Silver Nanoparticles on Kasumi 1 Cells

**Figure 8:**
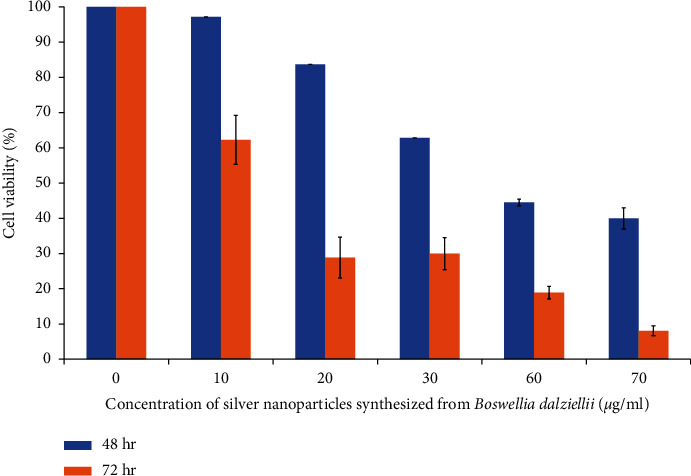
Antiproliferative effect of *B. dalzielii* AgNps on Kasumi-1 cell growth. The antiproliferative effect of the synthesized silver nanoparticles on Kasumi-1 leukemia cells was evaluated using PrestoBlue assay. As observed in the results, the nanoparticles inhibited the cancer cells in concentration and time dependent manners. The values are significantly different from one another at *p* < 0.05 as determined by one-way ANOVA.

The antiproliferative effect of the nanoparticles on Kasumi-1 leukemic cells was investigated using the PrestoBlue cell viability method, and the results are displayed in Figure 8. After 48-hour treatment, 10 *μ*g/ml of the nanoparticles inhibited just about 3% of the cell growth; however, as the concentration was increased the cancer cell population decreased, and 70 *μ*g/ml of the nanoparticles inhibited 60% of the cell growth which is a significant percentage. Expectedly, the inhibitory effect of the nanoparticles after 72-hour treatment was higher and more pronounced. After 72 hours, 10 *μ*g/ml of the nanoparticles inhibited 38% of the leukemic cell growth, and the cell growth significantly decreased as the concentration of the nanoparticles was raised. The highest concentration used (70 *μ*g/ml) inhibited 92% of the cell growth. The half minimal inhibitory concentrations (IC50s) that inhibited the Kasumi-1 cell growth are 49.5 *μ*g/ml and 13.25 *μ*g/ml at 48 hours and 72 hours, respectively. Therefore, the nanoparticles inhibited the proliferation of the Kasumi-1 leukemic cells in concentration and time dependent manners. The results showed that the nanoparticles inhibited the growth of the Kasumi-1 cells, and they are comparable to the results obtained for the inhibitory effects of other synthesized nanoparticles, substances, and chemicals [1, 41–45]. Silver nanoparticles synthesized from *Moringa oleifera* leaf extract inhibited AML Kasumi-1 cell growth with IC50 of 7.5 *μ*g/ml after 72-hour treatment [46]. Furthermore, PLGA-antiCD44-PTL nanoparticles inhibited the Kasumi-1 leukemic cells by decreasing its viability by 40% [47]. In addition, Li et al. [48] reported that CD33-targeted lipid nanoparticles (aCD33LNs) they synthesized effectively delivered GTI-2040 to the intended target to inhibit the proliferation of Kasumi-1 cells. The effect of the nanoparticles (aCD33LN/GTI-2040) was 15-fold more effective than the known antileukemic drug, Ara-C. Therefore, based on these observations, the synthesized nanoparticles are potential antileukemic cancer agents.

### 3.6. Effect of the Silver Nanoparticles on the Cell Cycle Progression of Kasumi 1 Cells

**Figure 9:**
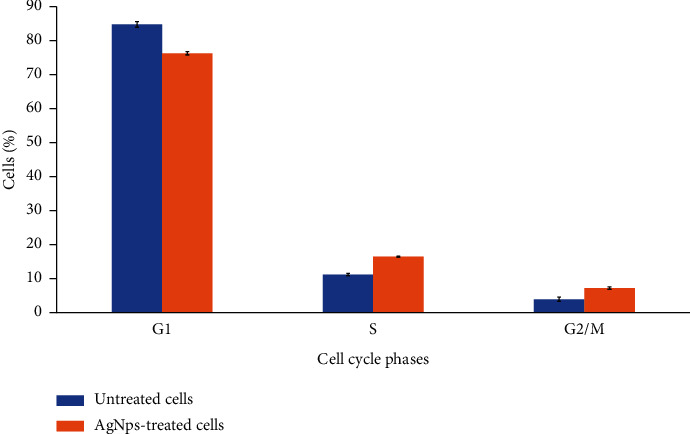
The effect of *B. dalzielii* AgNps on the cell cycle progression in Kasumi-1 cells. The effect of the synthesized silver nanoparticles on cell cycle progression of Kasumi-1 cells was determined. The results as displayed showed that the nanoparticles arrested the cell cycle progression of cancer cells at S and G2/M phases. Values plotted are means of four independent replicates. The values are significantly different from one another at *p* < 0.05 as determined by one-way ANOVA.

The response of the Kasumi-1 cell cycle progression to the synthesized nanoparticles was investigated by flow cytometry analysis, and the results are presented in Figure 9. Most of the control cells (untreated) were at the G1 phase (85%) while the rest were at S (11%) and G2/M (4%) phases, respectively. The discovery of most of the untreated cells (control) at G1 is an indication that the cells were actively viable, proliferating, and healthy [49]. In contrast, the distribution of the cells in the cell cycle phases was disrupted when they were treated with the synthesized nanoparticles which led to the significant accumulation and arrest of the cells at S and G2/M phases with subsequent decrease in the cells at G1 phase. The percentages of the cells at G1 phase decreased to 76%, and the percentages at S and G2/M phases increased to 16% and 7%, respectively. The observed arrest at S phase indicates that there is damage in the synthesis and replication of the cell's DNA, hence resulting in the subsequent arrest at G2/M phase to prevent such deficient cells from entering mitosis and proceeding in the cell cycle process [50]. This will cause the proliferation of the cells to be retarded and could also lead to cell death [51]. In a similar study, Khor et al. [46] also found that the silver nanoparticles that were synthesized from *M. oleifera* leaf extract induced S phase cell cycle arrest in Kasumi-1 cells.

## 4. Conclusion

Herein, we investigated the antileukemia effect of silver nanoparticles that were synthesized from the electron-rich metabolites of stem bark extract of *B. dalzielii* plant. Interestingly, the synthesized silver nanoparticles were found to have substantial antioxidant capacity. More importantly, the nanoparticles inhibited the growth of Kasumi-1 leukemia cells in concentration and time dependent manners. Then, the nanoparticles induced Kasumi-1 cell cycle arrests at S and G2/M phases. Thus, the synthesized silver nanoparticles are potential antileukemia agents. However, *in vivo* studies are needed to determine the toxicity and the antileukemia effect of the nanoparticles in animal subjects.

## Data Availability

The data used to support the findings of this study are available from the corresponding author upon request.
